# Supplementary material for: Modeling glioblastoma heterogeneity as a dynamic network of cell states
Source: Mol Syst Biol. 2021 Sep 16;17(9):e10105. doi: 10.15252/msb.202010105 (PMC8444284; doi:10.15252/msb.202010105)
Supplement: Supplementary file 6 — Source Data for Figure 5 [file MSB-17-e10105-s004.zip › Figure5A_sourcedata/GSEA_3071/nosigdb_stateI.GseaPreranked.1622795145664/RICHARDS_NOWAKOWSKI_SCIENCE_2017_MGE_IPC2_UPREG.html]

Details for gene set RICHARDS\_NOWAKOWSKI\_SCIENCE\_2017\_MGE\_IPC2\_UPREG[GSEA]

|  || Dataset | state13071 |
| Phenotype | NoPhenotypeAvailable |
| Upregulated in class | na\_neg |
| GeneSet | RICHARDS\_NOWAKOWSKI\_SCIENCE\_2017\_MGE\_IPC2\_UPREG |
| Enrichment Score (ES) | -0.58460367 |
| Normalized Enrichment Score (NES) | -3.651586 |
| Nominal p-value | 0.0 |
| FDR q-value | 0.0 |
| FWER p-Value | 0.0 |
Table: GSEA Results Summary

  

Fig 1: Enrichment plot: RICHARDS\_NOWAKOWSKI\_SCIENCE\_2017\_MGE\_IPC2\_UPREG      
 Profile of the Running ES Score & Positions of GeneSet Members on the Rank Ordered List

  

| PROBE | GENE SYMBOL | GENE\_TITLE | RANK IN GENE LIST | RANK METRIC SCORE | RUNNING ES | CORE ENRICHMENT || 1 | ARL4C |  |  | 108 | 1.062 | -0.0293 | No |
| 2 | STK17B |  |  | 274 | 0.707 | -0.0783 | No |
| 3 | BAHCC1 |  |  | 335 | 0.638 | -0.0944 | No |
| 4 | TIAM1 |  |  | 341 | 0.636 | -0.0931 | No |
| 5 | HERPUD1 |  |  | 436 | 0.561 | -0.1203 | No |
| 6 | MYO1B |  |  | 441 | 0.556 | -0.1191 | No |
| 7 | DOCK11 |  |  | 520 | 0.495 | -0.1415 | No |
| 8 | PDLIM3 |  |  | 547 | 0.481 | -0.1475 | No |
| 9 | IGF2BP1 |  |  | 591 | 0.457 | -0.1591 | No |
| 10 | UTRN |  |  | 604 | 0.449 | -0.1608 | No |
| 11 | PIP4K2A |  |  | 736 | 0.401 | -0.2005 | No |
| 12 | CLIC4 |  |  | 800 | 0.382 | -0.2187 | No |
| 13 | DST |  |  | 804 | 0.380 | -0.2179 | No |
| 14 | SLC38A1 |  |  | 1021 | 0.323 | -0.2848 | No |
| 15 | IDH2 |  |  | 1324 | 0.264 | -0.3791 | No |
| 16 | MSH2 |  |  | 1438 | -0.252 | -0.4137 | No |
| 17 | CEP152 |  |  | 1446 | -0.253 | -0.4148 | No |
| 18 | RALY |  |  | 1448 | -0.253 | -0.4140 | No |
| 19 | CTCF |  |  | 1450 | -0.253 | -0.4131 | No |
| 20 | AASS |  |  | 1457 | -0.253 | -0.4139 | No |
| 21 | GAPDH |  |  | 1459 | -0.253 | -0.4131 | No |
| 22 | SRSF10 |  |  | 1489 | -0.256 | -0.4211 | No |
| 23 | HNRNPH1 |  |  | 1500 | -0.257 | -0.4231 | No |
| 24 | ZGRF1 |  |  | 1505 | -0.258 | -0.4232 | No |
| 25 | RBBP4 |  |  | 1549 | -0.262 | -0.4356 | No |
| 26 | POLE2 |  |  | 1560 | -0.263 | -0.4376 | No |
| 27 | ARL6IP6 |  |  | 1577 | -0.265 | -0.4414 | No |
| 28 | TTK |  |  | 1620 | -0.270 | -0.4535 | No |
| 29 | MPHOSPH9 |  |  | 1623 | -0.270 | -0.4529 | No |
| 30 | STAG1 |  |  | 1624 | -0.270 | -0.4517 | No |
| 31 | SAE1 |  |  | 1638 | -0.273 | -0.4545 | No |
| 32 | SMC3 |  |  | 1650 | -0.274 | -0.4568 | No |
| 33 | BUB3 |  |  | 1687 | -0.278 | -0.4669 | No |
| 34 | YBX1 |  |  | 1725 | -0.283 | -0.4773 | No |
| 35 | KIF2C |  |  | 1735 | -0.284 | -0.4789 | No |
| 36 | SACS |  |  | 1738 | -0.285 | -0.4782 | No |
| 37 | PSMC3 |  |  | 1752 | -0.287 | -0.4810 | No |
| 38 | CCNB2 |  |  | 1757 | -0.288 | -0.4810 | No |
| 39 | MIS18BP1 |  |  | 1764 | -0.289 | -0.4816 | No |
| 40 | CENPJ |  |  | 1768 | -0.290 | -0.4812 | No |
| 41 | EXOSC9 |  |  | 1777 | -0.291 | -0.4824 | No |
| 42 | APOLD1 |  |  | 1781 | -0.291 | -0.4820 | No |
| 43 | PIK3R3 |  |  | 1797 | -0.293 | -0.4854 | No |
| 44 | HNRNPA2B1 |  |  | 1819 | -0.296 | -0.4907 | No |
| 45 | TROAP |  |  | 1821 | -0.296 | -0.4897 | No |
| 46 | CASP8AP2 |  |  | 1857 | -0.300 | -0.4994 | No |
| 47 | CENPQ |  |  | 1860 | -0.300 | -0.4987 | No |
| 48 | TTF2 |  |  | 1873 | -0.301 | -0.5011 | No |
| 49 | OSBPL11 |  |  | 1889 | -0.303 | -0.5045 | No |
| 50 | CHIC2 |  |  | 1910 | -0.307 | -0.5094 | No |
| 51 | KPNA2 |  |  | 1944 | -0.312 | -0.5185 | No |
| 52 | ZNF92 |  |  | 1962 | -0.313 | -0.5224 | No |
| 53 | OPA1 |  |  | 2014 | -0.319 | -0.5371 | No |
| 54 | PARPBP |  |  | 2026 | -0.322 | -0.5391 | No |
| 55 | SOX6 |  |  | 2032 | -0.323 | -0.5392 | No |
| 56 | EFS |  |  | 2038 | -0.323 | -0.5393 | No |
| 57 | DDX39A |  |  | 2043 | -0.324 | -0.5391 | No |
| 58 | ACYP1 |  |  | 2052 | -0.326 | -0.5402 | No |
| 59 | DNA2 |  |  | 2057 | -0.327 | -0.5400 | No |
| 60 | SFMBT1 |  |  | 2061 | -0.327 | -0.5394 | No |
| 61 | CCDC15 |  |  | 2080 | -0.331 | -0.5436 | No |
| 62 | E2F7 |  |  | 2081 | -0.331 | -0.5421 | No |
| 63 | CHEK1 |  |  | 2108 | -0.336 | -0.5488 | No |
| 64 | C18orf54 |  |  | 2128 | -0.339 | -0.5533 | No |
| 65 | ZNF367 |  |  | 2131 | -0.339 | -0.5524 | No |
| 66 | CORO1C |  |  | 2136 | -0.340 | -0.5521 | No |
| 67 | HNRNPDL |  |  | 2142 | -0.342 | -0.5521 | No |
| 68 | PLK4 |  |  | 2167 | -0.346 | -0.5581 | No |
| 69 | NGLY1 |  |  | 2181 | -0.348 | -0.5607 | No |
| 70 | YEATS4 |  |  | 2187 | -0.349 | -0.5607 | No |
| 71 | HNRNPL |  |  | 2190 | -0.350 | -0.5597 | No |
| 72 | BRD7 |  |  | 2193 | -0.350 | -0.5588 | No |
| 73 | DLEU2 |  |  | 2196 | -0.351 | -0.5578 | No |
| 74 | GLCCI1 |  |  | 2212 | -0.356 | -0.5609 | No |
| 75 | SMC1A |  |  | 2240 | -0.361 | -0.5678 | No |
| 76 | HMGN1 |  |  | 2248 | -0.363 | -0.5684 | No |
| 77 | POLQ |  |  | 2253 | -0.364 | -0.5680 | No |
| 78 | NUCKS1 |  |  | 2278 | -0.368 | -0.5739 | No |
| 79 | DEPDC1B |  |  | 2288 | -0.370 | -0.5751 | No |
| 80 | TUBB4B |  |  | 2291 | -0.370 | -0.5740 | No |
| 81 | FUS |  |  | 2300 | -0.373 | -0.5749 | No |
| 82 | MSI2 |  |  | 2314 | -0.375 | -0.5773 | No |
| 83 | TSC22D2 |  |  | 2328 | -0.379 | -0.5797 | No |
| 84 | CKS1B |  |  | 2332 | -0.380 | -0.5789 | No |
| 85 | BUB1B |  |  | 2335 | -0.381 | -0.5778 | No |
| 86 | CKAP2 |  |  | 2357 | -0.385 | -0.5827 | No |
| 87 | PSIP1 |  |  | 2364 | -0.386 | -0.5828 | Yes |
| 88 | CCNF |  |  | 2370 | -0.387 | -0.5827 | Yes |
| 89 | STIL |  |  | 2374 | -0.388 | -0.5819 | Yes |
| 90 | POLA1 |  |  | 2376 | -0.388 | -0.5804 | Yes |
| 91 | H3F3A |  |  | 2377 | -0.388 | -0.5786 | Yes |
| 92 | ABAT |  |  | 2383 | -0.390 | -0.5785 | Yes |
| 93 | HAUS6 |  |  | 2388 | -0.390 | -0.5779 | Yes |
| 94 | RDX |  |  | 2399 | -0.392 | -0.5793 | Yes |
| 95 | CENPW |  |  | 2402 | -0.393 | -0.5782 | Yes |
| 96 | UHRF1 |  |  | 2404 | -0.393 | -0.5767 | Yes |
| 97 | PRIM2 |  |  | 2409 | -0.394 | -0.5762 | Yes |
| 98 | BUB1 |  |  | 2416 | -0.396 | -0.5763 | Yes |
| 99 | HAUS1 |  |  | 2423 | -0.397 | -0.5764 | Yes |
| 100 | WEE1 |  |  | 2426 | -0.397 | -0.5752 | Yes |
| 101 | H2AFV |  |  | 2431 | -0.398 | -0.5747 | Yes |
| 102 | DSEL |  |  | 2432 | -0.398 | -0.5728 | Yes |
| 103 | HNRNPU |  |  | 2434 | -0.398 | -0.5714 | Yes |
| 104 | KCTD9 |  |  | 2438 | -0.399 | -0.5705 | Yes |
| 105 | ZNF738 |  |  | 2441 | -0.400 | -0.5693 | Yes |
| 106 | NCAPH2 |  |  | 2443 | -0.400 | -0.5678 | Yes |
| 107 | HNRNPA1 |  |  | 2455 | -0.402 | -0.5695 | Yes |
| 108 | CENPO |  |  | 2460 | -0.404 | -0.5689 | Yes |
| 109 | ARHGEF26 |  |  | 2465 | -0.404 | -0.5683 | Yes |
| 110 | CKS2 |  |  | 2466 | -0.404 | -0.5665 | Yes |
| 111 | BLM |  |  | 2469 | -0.404 | -0.5653 | Yes |
| 112 | KIF11 |  |  | 2488 | -0.410 | -0.5691 | Yes |
| 113 | PIP5K1B |  |  | 2492 | -0.411 | -0.5682 | Yes |
| 114 | HAUS8 |  |  | 2494 | -0.412 | -0.5666 | Yes |
| 115 | CENPL |  |  | 2497 | -0.413 | -0.5654 | Yes |
| 116 | ZNF519 |  |  | 2504 | -0.416 | -0.5654 | Yes |
| 117 | SPAG5 |  |  | 2519 | -0.418 | -0.5679 | Yes |
| 118 | RACGAP1 |  |  | 2522 | -0.419 | -0.5667 | Yes |
| 119 | KCNJ2 |  |  | 2528 | -0.422 | -0.5663 | Yes |
| 120 | BAZ1B |  |  | 2538 | -0.425 | -0.5672 | Yes |
| 121 | CDC20 |  |  | 2540 | -0.425 | -0.5656 | Yes |
| 122 | FIGN |  |  | 2543 | -0.426 | -0.5643 | Yes |
| 123 | PAICS |  |  | 2545 | -0.426 | -0.5627 | Yes |
| 124 | PCM1 |  |  | 2555 | -0.428 | -0.5636 | Yes |
| 125 | NCAPD2 |  |  | 2563 | -0.431 | -0.5639 | Yes |
| 126 | CDK5RAP2 |  |  | 2564 | -0.431 | -0.5619 | Yes |
| 127 | RSRC1 |  |  | 2571 | -0.433 | -0.5618 | Yes |
| 128 | H2AFZ |  |  | 2572 | -0.433 | -0.5599 | Yes |
| 129 | TMEM106C |  |  | 2592 | -0.437 | -0.5639 | Yes |
| 130 | ANP32B |  |  | 2593 | -0.438 | -0.5619 | Yes |
| 131 | PCDH17 |  |  | 2600 | -0.439 | -0.5618 | Yes |
| 132 | PBRM1 |  |  | 2603 | -0.440 | -0.5604 | Yes |
| 133 | CIT |  |  | 2604 | -0.440 | -0.5584 | Yes |
| 134 | UBR7 |  |  | 2611 | -0.442 | -0.5583 | Yes |
| 135 | KNTC1 |  |  | 2613 | -0.443 | -0.5566 | Yes |
| 136 | RMI1 |  |  | 2616 | -0.444 | -0.5552 | Yes |
| 137 | NCAPG2 |  |  | 2621 | -0.445 | -0.5545 | Yes |
| 138 | FANCB |  |  | 2623 | -0.446 | -0.5528 | Yes |
| 139 | SRSF3 |  |  | 2631 | -0.447 | -0.5529 | Yes |
| 140 | HIRIP3 |  |  | 2635 | -0.449 | -0.5519 | Yes |
| 141 | LINC01224 |  |  | 2637 | -0.449 | -0.5501 | Yes |
| 142 | DUT |  |  | 2640 | -0.450 | -0.5487 | Yes |
| 143 | MASTL |  |  | 2646 | -0.452 | -0.5482 | Yes |
| 144 | EXOSC8 |  |  | 2659 | -0.456 | -0.5500 | Yes |
| 145 | CCNA2 |  |  | 2667 | -0.462 | -0.5501 | Yes |
| 146 | RFWD3 |  |  | 2668 | -0.462 | -0.5480 | Yes |
| 147 | SKA2 |  |  | 2672 | -0.464 | -0.5468 | Yes |
| 148 | MND1 |  |  | 2682 | -0.468 | -0.5475 | Yes |
| 149 | KIF22 |  |  | 2683 | -0.468 | -0.5454 | Yes |
| 150 | TOP2B |  |  | 2686 | -0.469 | -0.5439 | Yes |
| 151 | PCNP |  |  | 2691 | -0.470 | -0.5430 | Yes |
| 152 | CDCA8 |  |  | 2695 | -0.470 | -0.5419 | Yes |
| 153 | MMS22L |  |  | 2698 | -0.471 | -0.5403 | Yes |
| 154 | MCM6 |  |  | 2699 | -0.471 | -0.5382 | Yes |
| 155 | ANLN |  |  | 2702 | -0.472 | -0.5367 | Yes |
| 156 | HAT1 |  |  | 2703 | -0.472 | -0.5346 | Yes |
| 157 | PRC1 |  |  | 2705 | -0.472 | -0.5327 | Yes |
| 158 | FANCD2 |  |  | 2707 | -0.474 | -0.5309 | Yes |
| 159 | CENPN |  |  | 2708 | -0.474 | -0.5287 | Yes |
| 160 | MELK |  |  | 2709 | -0.474 | -0.5266 | Yes |
| 161 | NDC80 |  |  | 2723 | -0.478 | -0.5285 | Yes |
| 162 | EXO1 |  |  | 2728 | -0.479 | -0.5276 | Yes |
| 163 | PDZRN3 |  |  | 2731 | -0.480 | -0.5260 | Yes |
| 164 | BRIP1 |  |  | 2739 | -0.482 | -0.5261 | Yes |
| 165 | HIST1H2BH |  |  | 2748 | -0.485 | -0.5264 | Yes |
| 166 | TIMELESS |  |  | 2752 | -0.487 | -0.5251 | Yes |
| 167 | CENPF |  |  | 2761 | -0.490 | -0.5254 | Yes |
| 168 | BRCA2 |  |  | 2772 | -0.495 | -0.5264 | Yes |
| 169 | NUF2 |  |  | 2781 | -0.497 | -0.5266 | Yes |
| 170 | TUBA1B |  |  | 2785 | -0.498 | -0.5253 | Yes |
| 171 | SKA1 |  |  | 2787 | -0.498 | -0.5234 | Yes |
| 172 | HIST1H3D |  |  | 2790 | -0.499 | -0.5217 | Yes |
| 173 | MGME1 |  |  | 2797 | -0.500 | -0.5213 | Yes |
| 174 | DTYMK |  |  | 2798 | -0.501 | -0.5191 | Yes |
| 175 | WDR76 |  |  | 2800 | -0.501 | -0.5171 | Yes |
| 176 | RAD18 |  |  | 2802 | -0.501 | -0.5151 | Yes |
| 177 | HMGB1 |  |  | 2806 | -0.503 | -0.5138 | Yes |
| 178 | RBL1 |  |  | 2808 | -0.503 | -0.5118 | Yes |
| 179 | COX8A |  |  | 2819 | -0.509 | -0.5127 | Yes |
| 180 | ARL6IP1 |  |  | 2823 | -0.510 | -0.5113 | Yes |
| 181 | CDCA3 |  |  | 2829 | -0.513 | -0.5106 | Yes |
| 182 | SPC24 |  |  | 2836 | -0.514 | -0.5101 | Yes |
| 183 | HMGB3 |  |  | 2839 | -0.516 | -0.5084 | Yes |
| 184 | TOP2A |  |  | 2842 | -0.517 | -0.5067 | Yes |
| 185 | ZNF43 |  |  | 2844 | -0.517 | -0.5047 | Yes |
| 186 | E2F8 |  |  | 2847 | -0.519 | -0.5029 | Yes |
| 187 | LIG1 |  |  | 2848 | -0.520 | -0.5006 | Yes |
| 188 | WDHD1 |  |  | 2853 | -0.522 | -0.4995 | Yes |
| 189 | CEP97 |  |  | 2856 | -0.522 | -0.4977 | Yes |
| 190 | CKAP2L |  |  | 2857 | -0.523 | -0.4954 | Yes |
| 191 | ECT2 |  |  | 2858 | -0.523 | -0.4930 | Yes |
| 192 | NUDT1 |  |  | 2859 | -0.524 | -0.4906 | Yes |
| 193 | CDK2 |  |  | 2863 | -0.526 | -0.4892 | Yes |
| 194 | RAD21 |  |  | 2866 | -0.527 | -0.4874 | Yes |
| 195 | NUP62 |  |  | 2867 | -0.527 | -0.4850 | Yes |
| 196 | HJURP |  |  | 2869 | -0.527 | -0.4829 | Yes |
| 197 | ANP32E |  |  | 2877 | -0.530 | -0.4827 | Yes |
| 198 | PTTG1 |  |  | 2895 | -0.540 | -0.4856 | Yes |
| 199 | ARX |  |  | 2898 | -0.542 | -0.4838 | Yes |
| 200 | SPECC1 |  |  | 2909 | -0.546 | -0.4845 | Yes |
| 201 | FAM111A |  |  | 2917 | -0.552 | -0.4842 | Yes |
| 202 | BEST3 |  |  | 2918 | -0.553 | -0.4817 | Yes |
| 203 | ARHGAP11A |  |  | 2921 | -0.556 | -0.4798 | Yes |
| 204 | ARL4D |  |  | 2922 | -0.558 | -0.4773 | Yes |
| 205 | TRA2B |  |  | 2933 | -0.563 | -0.4779 | Yes |
| 206 | ATAD5 |  |  | 2935 | -0.563 | -0.4756 | Yes |
| 207 | PSRC1 |  |  | 2944 | -0.569 | -0.4756 | Yes |
| 208 | OLA1 |  |  | 2946 | -0.569 | -0.4733 | Yes |
| 209 | MIS18A |  |  | 2947 | -0.570 | -0.4707 | Yes |
| 210 | DSN1 |  |  | 2949 | -0.571 | -0.4684 | Yes |
| 211 | MAD2L2 |  |  | 2952 | -0.573 | -0.4665 | Yes |
| 212 | KIF4A |  |  | 2953 | -0.574 | -0.4638 | Yes |
| 213 | PBX3 |  |  | 2959 | -0.578 | -0.4628 | Yes |
| 214 | KIF23 |  |  | 2961 | -0.579 | -0.4605 | Yes |
| 215 | KLHL13 |  |  | 2965 | -0.580 | -0.4588 | Yes |
| 216 | H1FX |  |  | 2968 | -0.581 | -0.4568 | Yes |
| 217 | NCAPH |  |  | 2974 | -0.585 | -0.4557 | Yes |
| 218 | VRK1 |  |  | 2983 | -0.592 | -0.4556 | Yes |
| 219 | SMC2 |  |  | 2984 | -0.592 | -0.4529 | Yes |
| 220 | SKA3 |  |  | 2985 | -0.592 | -0.4502 | Yes |
| 221 | ILDR2 |  |  | 2990 | -0.595 | -0.4487 | Yes |
| 222 | TPX2 |  |  | 3006 | -0.606 | -0.4507 | Yes |
| 223 | RTKN2 |  |  | 3020 | -0.613 | -0.4521 | Yes |
| 224 | ZEB2 |  |  | 3031 | -0.620 | -0.4524 | Yes |
| 225 | TOPBP1 |  |  | 3033 | -0.621 | -0.4499 | Yes |
| 226 | DNMT1 |  |  | 3035 | -0.622 | -0.4474 | Yes |
| 227 | LSM3 |  |  | 3037 | -0.625 | -0.4449 | Yes |
| 228 | RBBP8 |  |  | 3053 | -0.637 | -0.4467 | Yes |
| 229 | AURKB |  |  | 3056 | -0.639 | -0.4444 | Yes |
| 230 | NCAPD3 |  |  | 3064 | -0.646 | -0.4437 | Yes |
| 231 | ALYREF |  |  | 3068 | -0.649 | -0.4417 | Yes |
| 232 | FANCI |  |  | 3074 | -0.656 | -0.4403 | Yes |
| 233 | C21orf58 |  |  | 3078 | -0.659 | -0.4383 | Yes |
| 234 | FBXW7 |  |  | 3082 | -0.662 | -0.4362 | Yes |
| 235 | GTSE1 |  |  | 3083 | -0.663 | -0.4332 | Yes |
| 236 | ZNF704 |  |  | 3086 | -0.665 | -0.4308 | Yes |
| 237 | TMPO |  |  | 3088 | -0.666 | -0.4281 | Yes |
| 238 | SAP30 |  |  | 3096 | -0.671 | -0.4273 | Yes |
| 239 | MAD2L1 |  |  | 3099 | -0.675 | -0.4248 | Yes |
| 240 | NASP |  |  | 3106 | -0.682 | -0.4236 | Yes |
| 241 | RFC3 |  |  | 3108 | -0.683 | -0.4208 | Yes |
| 242 | KIFC1 |  |  | 3110 | -0.684 | -0.4180 | Yes |
| 243 | DBF4 |  |  | 3117 | -0.690 | -0.4168 | Yes |
| 244 | MYBL1 |  |  | 3119 | -0.691 | -0.4140 | Yes |
| 245 | LMNB1 |  |  | 3121 | -0.692 | -0.4111 | Yes |
| 246 | POLD3 |  |  | 3122 | -0.695 | -0.4080 | Yes |
| 247 | PRR11 |  |  | 3125 | -0.698 | -0.4054 | Yes |
| 248 | SMC4 |  |  | 3126 | -0.699 | -0.4023 | Yes |
| 249 | CENPK |  |  | 3127 | -0.700 | -0.3991 | Yes |
| 250 | RFC4 |  |  | 3129 | -0.701 | -0.3962 | Yes |
| 251 | MCM5 |  |  | 3132 | -0.706 | -0.3936 | Yes |
| 252 | MEIS1 |  |  | 3135 | -0.711 | -0.3910 | Yes |
| 253 | CCDC88A |  |  | 3138 | -0.714 | -0.3884 | Yes |
| 254 | GMNN |  |  | 3140 | -0.719 | -0.3855 | Yes |
| 255 | KIF15 |  |  | 3142 | -0.723 | -0.3825 | Yes |
| 256 | BCL11B |  |  | 3146 | -0.725 | -0.3802 | Yes |
| 257 | BTG3 |  |  | 3148 | -0.727 | -0.3772 | Yes |
| 258 | CDK1 |  |  | 3149 | -0.727 | -0.3739 | Yes |
| 259 | AGO2 |  |  | 3153 | -0.732 | -0.3715 | Yes |
| 260 | RNF168 |  |  | 3154 | -0.734 | -0.3681 | Yes |
| 261 | HNRNPD |  |  | 3157 | -0.738 | -0.3654 | Yes |
| 262 | MKI67 |  |  | 3158 | -0.738 | -0.3621 | Yes |
| 263 | ORC6 |  |  | 3159 | -0.738 | -0.3587 | Yes |
| 264 | PRIM1 |  |  | 3160 | -0.739 | -0.3554 | Yes |
| 265 | TK1 |  |  | 3162 | -0.745 | -0.3523 | Yes |
| 266 | RNASEH2B |  |  | 3166 | -0.747 | -0.3498 | Yes |
| 267 | STMN1 |  |  | 3171 | -0.751 | -0.3477 | Yes |
| 268 | MCM8 |  |  | 3178 | -0.758 | -0.3461 | Yes |
| 269 | MCM3 |  |  | 3182 | -0.761 | -0.3436 | Yes |
| 270 | USP1 |  |  | 3183 | -0.762 | -0.3402 | Yes |
| 271 | BRCA1 |  |  | 3185 | -0.765 | -0.3370 | Yes |
| 272 | CDCA2 |  |  | 3189 | -0.767 | -0.3345 | Yes |
| 273 | MAGI1 |  |  | 3193 | -0.771 | -0.3319 | Yes |
| 274 | XRCC2 |  |  | 3197 | -0.777 | -0.3293 | Yes |
| 275 | BARD1 |  |  | 3201 | -0.783 | -0.3267 | Yes |
| 276 | CDCA7 |  |  | 3203 | -0.785 | -0.3235 | Yes |
| 277 | BIRC5 |  |  | 3208 | -0.793 | -0.3211 | Yes |
| 278 | GAD1 |  |  | 3211 | -0.796 | -0.3181 | Yes |
| 279 | FBXO5 |  |  | 3212 | -0.797 | -0.3145 | Yes |
| 280 | DIAPH3 |  |  | 3213 | -0.800 | -0.3109 | Yes |
| 281 | RRM1 |  |  | 3215 | -0.801 | -0.3076 | Yes |
| 282 | SPC25 |  |  | 3219 | -0.806 | -0.3048 | Yes |
| 283 | DLX6 |  |  | 3223 | -0.810 | -0.3021 | Yes |
| 284 | MDK |  |  | 3224 | -0.811 | -0.2984 | Yes |
| 285 | UBE2C |  |  | 3225 | -0.812 | -0.2947 | Yes |
| 286 | SYNE2 |  |  | 3231 | -0.820 | -0.2926 | Yes |
| 287 | NUSAP1 |  |  | 3237 | -0.825 | -0.2904 | Yes |
| 288 | FOXM1 |  |  | 3238 | -0.826 | -0.2867 | Yes |
| 289 | TMSB15A |  |  | 3241 | -0.833 | -0.2835 | Yes |
| 290 | HMGN2 |  |  | 3242 | -0.833 | -0.2797 | Yes |
| 291 | RFC2 |  |  | 3245 | -0.839 | -0.2765 | Yes |
| 292 | DTL |  |  | 3249 | -0.848 | -0.2736 | Yes |
| 293 | PFN2 |  |  | 3252 | -0.852 | -0.2704 | Yes |
| 294 | NRXN3 |  |  | 3254 | -0.854 | -0.2668 | Yes |
| 295 | LCORL |  |  | 3256 | -0.859 | -0.2632 | Yes |
| 296 | CENPM |  |  | 3269 | -0.881 | -0.2630 | Yes |
| 297 | CHAF1A |  |  | 3270 | -0.882 | -0.2590 | Yes |
| 298 | CDCA5 |  |  | 3273 | -0.885 | -0.2556 | Yes |
| 299 | CCNE2 |  |  | 3276 | -0.889 | -0.2522 | Yes |
| 300 | FEN1 |  |  | 3278 | -0.890 | -0.2485 | Yes |
| 301 | GAS2L3 |  |  | 3283 | -0.899 | -0.2457 | Yes |
| 302 | HELLS |  |  | 3284 | -0.901 | -0.2416 | Yes |
| 303 | CDKN3 |  |  | 3293 | -0.916 | -0.2399 | Yes |
| 304 | ZWINT |  |  | 3296 | -0.925 | -0.2364 | Yes |
| 305 | FBLN1 |  |  | 3317 | -0.954 | -0.2384 | Yes |
| 306 | PCNA |  |  | 3319 | -0.963 | -0.2343 | Yes |
| 307 | UBE2T |  |  | 3330 | -0.979 | -0.2330 | Yes |
| 308 | MYBL2 |  |  | 3340 | -1.006 | -0.2313 | Yes |
| 309 | MCM4 |  |  | 3341 | -1.006 | -0.2267 | Yes |
| 310 | EZH2 |  |  | 3342 | -1.015 | -0.2221 | Yes |
| 311 | DEK |  |  | 3344 | -1.025 | -0.2178 | Yes |
| 312 | RAD51AP1 |  |  | 3345 | -1.030 | -0.2131 | Yes |
| 313 | LSAMP |  |  | 3346 | -1.033 | -0.2084 | Yes |
| 314 | PROM1 |  |  | 3347 | -1.035 | -0.2037 | Yes |
| 315 | PBK |  |  | 3354 | -1.046 | -0.2008 | Yes |
| 316 | MCM10 |  |  | 3356 | -1.050 | -0.1964 | Yes |
| 317 | SP9 |  |  | 3358 | -1.060 | -0.1919 | Yes |
| 318 | CDK6 |  |  | 3366 | -1.085 | -0.1891 | Yes |
| 319 | ESCO2 |  |  | 3367 | -1.088 | -0.1842 | Yes |
| 320 | ASAP1 |  |  | 3369 | -1.090 | -0.1795 | Yes |
| 321 | NCAPG |  |  | 3370 | -1.098 | -0.1746 | Yes |
| 322 | ZBTB20 |  |  | 3373 | -1.109 | -0.1701 | Yes |
| 323 | RGS12 |  |  | 3375 | -1.111 | -0.1654 | Yes |
| 324 | MCM7 |  |  | 3384 | -1.147 | -0.1627 | Yes |
| 325 | GINS2 |  |  | 3386 | -1.156 | -0.1578 | Yes |
| 326 | DHFR |  |  | 3387 | -1.159 | -0.1525 | Yes |
| 327 | CHD7 |  |  | 3389 | -1.167 | -0.1475 | Yes |
| 328 | HMGB2 |  |  | 3390 | -1.184 | -0.1422 | Yes |
| 329 | CLSPN |  |  | 3391 | -1.184 | -0.1368 | Yes |
| 330 | SOX2 |  |  | 3394 | -1.198 | -0.1320 | Yes |
| 331 | RAB3IP |  |  | 3395 | -1.198 | -0.1265 | Yes |
| 332 | CENPU |  |  | 3398 | -1.230 | -0.1215 | Yes |
| 333 | DCLK2 |  |  | 3400 | -1.237 | -0.1162 | Yes |
| 334 | HIST1H1E |  |  | 3401 | -1.237 | -0.1106 | Yes |
| 335 | FAM111B |  |  | 3402 | -1.238 | -0.1050 | Yes |
| 336 | NEDD4L |  |  | 3406 | -1.247 | -0.1003 | Yes |
| 337 | ATAD2 |  |  | 3415 | -1.278 | -0.0970 | Yes |
| 338 | NCAM1 |  |  | 3416 | -1.284 | -0.0912 | Yes |
| 339 | RRM2 |  |  | 3420 | -1.297 | -0.0862 | Yes |
| 340 | HIST1H1C |  |  | 3427 | -1.335 | -0.0820 | Yes |
| 341 | TYMS |  |  | 3436 | -1.391 | -0.0782 | Yes |
| 342 | CDH2 |  |  | 3439 | -1.439 | -0.0723 | Yes |
| 343 | DLX1 |  |  | 3455 | -1.622 | -0.0697 | Yes |
| 344 | HIST1H1D |  |  | 3466 | -1.743 | -0.0649 | Yes |
| 345 | HIST1H1B |  |  | 3467 | -1.770 | -0.0569 | Yes |
| 346 | DLX2 |  |  | 3474 | -1.814 | -0.0506 | Yes |
| 347 | CDCA7L |  |  | 3481 | -1.878 | -0.0439 | Yes |
| 348 | INSM1 |  |  | 3483 | -1.929 | -0.0355 | Yes |
| 349 | ASCL1 |  |  | 3484 | -1.937 | -0.0267 | Yes |
| 350 | DLX5 |  |  | 3486 | -2.052 | -0.0176 | Yes |
| 351 | HIST1H4C |  |  | 3493 | -2.248 | -0.0093 | Yes |
| 352 | LMO1 |  |  | 3507 | -3.235 | 0.0013 | Yes |
Table: GSEA details [plain text format]

  

Fig 2: RICHARDS\_NOWAKOWSKI\_SCIENCE\_2017\_MGE\_IPC2\_UPREG: Random ES distribution      
 Gene set null distribution of ES for **RICHARDS\_NOWAKOWSKI\_SCIENCE\_2017\_MGE\_IPC2\_UPREG**
